# Risk-reducing strategies for women carrying brca1/2 mutations with a focus on prophylactic surgery

**DOI:** 10.1186/1472-6874-10-28

**Published:** 2010-10-20

**Authors:** Mohamed Salhab, Selina Bismohun, Kefah Mokbel

**Affiliations:** 1 St. George's University of London, London, UK; 2 London Breast Institute, The Princess Grace Hospital, 45 Nottingham Place, London, W1U 5NY, UK

## Abstract

**Background:**

Women who have inherited mutations in the BRCA1 or BRCA2 genes have substantially elevated risks of breast and ovarian cancer. Mutation carriers have various options, including extensive and regular surveillance, chemoprevention and risk-reducing surgery. The aim of this review is to provide an up-to-date analysis and to subsequently summarise the available literature in relation to risk-reducing strategies, with a keen focus on prophylactic surgery.

**Methods:**

The literature review is facilitated by Medline and PubMed databases. The cross-referencing of the obtained articles was used to identify other relevant studies.

**Results:**

Prophylactic surgery (bilateral mastectomy, bilateral salpingo-oophorectomy or a combination of both procedures) has proved to be the most effective risk-reducing strategy. There are no randomised controlled trials able to demonstrate the potential benefits or harms of prophylactic surgery; therefore, the evidence has been derived from retrospective and short follow-up prospective studies, in addition to hypothetical mathematical models.

Based on the current knowledge, it is reasonable to recommend prophylactic oophorectomy for BRCA1 or BRCA2 mutation carriers when childbearing is completed in order to reduce the risk of developing breast and ovarian cancer. In addition, women should be offered the options of rigorous breast surveillance, chemoprevention with anti-oestrogens--especially for carriers of BRCA2--or bilateral prophylactic mastectomy.

**Conclusion:**

The selection of the most appropriate risk-reducing strategy is not a straightforward task. The impact of risk-reducing strategies on cancer risk, survival, and overall quality of life are the key criteria considered for decision-making. Notably, various other factors should be taken into consideration when evaluating individual mutation carriers' individual circumstances, namely woman's age, morbidity, type of mutation, and individual preferences and expectations.

Although prospective randomised controlled trials concerned with examining the various interventions in relation to the woman's age and type of mutation are needed, randomisation is extremely difficult and rather deemed unethical given the current available evidence from retrospective studies.

## Background

Breast cancer remains the leading cause of death in women aged 40-55 years[1]. Sporadic breast cancer accounts for 70-80% of all cases [2]. It is estimated that 5-10% of all breast cancers and 25%-40% of breast cancers affecting women younger than 35 years of age are attributable to hereditary causes[2]. Furthermore 60%-70% of hereditary breast cancers seem to arise secondary to germline mutations in the BRCA1 and BRCA2 genes[3,4]. Notably, ovarian cancer is a relatively rare disease: it affects one in eighty women at some point during their life time[5]. Hereditary or familial ovarian cancer is linked to many germline mutations, such as PTEN (Cowden syndrome), TP53 (Li-Fraumeni Syndrome) and ATM (ataxia-teleangiectatica-mutated gene)[6]. However, BRCA1 and BRCA2 gene mutations are found to cause the majority of autosomal-dominant inherited ovarian cancer[6].

Histologically, BRCA1-related breast cancers are predominantly of the basal subtype, with predominant lymphocytic infiltration, and are often more aggressive and associated with negative prognostic factors, as characterised by numerous mitoses, pleomorphic pattern, poor differentiation and higher proliferation rates [7-11], as well as a negative oestrogen and progesterone receptors status [8,10,12]. Moreover, a lack of HER-2 expression was observed in breast cancer tumours of BRCA1 carriers[13]. On the other hand, tumours associated with BRCA2 mutations are of a luminal subtype, and tend to be of a positive oestrogen and progesterone receptors-status; negative HER-2, on the other hand, do not markedly differ from other hereditary or sporadic breast cancers[14]. DCIS is found to be equally as prevalent in patients who carry deleterious BRCA mutations as in high familial-risk women who are non-carriers, but ultimately occurs at an earlier age. Interestingly, BRCA mutations were found in a significant portion of women with DCIS who were presented for a hereditary risk assessment[15]. Notably, high-grade DCIS is also more common in BRCA1 mutation carriers than in those patients without any mutation[16]. Accordingly, it has been suggested that BRCA1/2-associated breast cancers progress through the same intermediate steps as sporadic breast cancers, and that DCIS should be considered as a part of the BRCA1/2 tumour spectrum[17].

Clinically, breast cancer is believed to begin at an earlier age in BRCA1 and BRCA2 gene mutation carriers compared with sporadic cases. The median age of breast cancer onset ranges from 40-50 years in BRCA1 and BRCA2 carriers compared with 60-70 years in sporadic cases[18,19]. Furthermore, breast tumours in BRCA1/2 carriers are more likely to be bilateral and affect the contra lateral breast at a later stage [8,12,19]. Furthermore, BRCA1/2 mutation carriers also have a higher rate of pregnancy-associated breast cancer before the age of 50[20,21].

Several studies have suggested no significant differences between BRCA-associated breast cancer and non-BRCA associated breast cancer in relapse-free, event-free, and overall survival [22-24]. On the other hand, other studies indicate that BRCA-associated breast cancer presents worse survival rates and overall prognosis [14,25-28]. Inconsistencies in previous studies may be due to limitations in study design: in particular, the studies contain small numbers of BRCA cases. Additionally, due to the onset occurring at an extremely early age of those patients with BRCA mutations, it was not always possible to obtain controls which were considered adequately matched for age. In addition, inconsistencies in previous studies were due to the examination of both BRCA1 and BRCA2 mutation groups together. Lee *et al*., for example, recently conducted a meta-analysis of 11 previously published articles [29]. The analysis shows that the BRCA1 mutation carriers have worse predicted overall survival rates (approximately 5 years) than non-carriers (HR = 1.94, 95% CI, 1.45-2.53). Furthermore, long-term overall survival (10 years or greater) was found to be worse in BRCA1 mutation carriers compared with non-carriers (HR = 1.33, 95% CI, 1.12-1.58). However, they indicate that there has been no significant heterogeneity observed across the studies.

Moreover, Lee *et al*. observes that short-term progression-free survival in BRCA1 carriers was worse than in non-BRCA carriers (HR = 1.54). Such observations were not witnessed in long-term progression survival. In addition, BRCA2 mutation was not observed to affect either short- or long-term survival rates, which may be attributed to the different carcinogenic pathways for BRCA1 and BRCA2 [29].

Most ovarian cancers associated with germ line BRCA mutations are diagnosed at a younger age and are ultimately determined as high-grade and advanced-stage serous carcinomas[30]. They have surgical and pathological characteristics similar to those of sporadic cancers. BRCA mutations do not seem to play a significant role in the development of mucinous or borderline ovarian tumours[21]. Moreover, BRCA1 germline mutation carriers are not only at risk of ovarian cancer, but also fallopian tube carcinoma and peritoneal papillary serous carcinoma[31]. Olivier *et al*. suggest that peritoneal papillary serous carcinoma risk amongst BRCA2 carriers is lower than amongst BRCA1 carriers[31]. Furthermore, hereditary ovarian cancer has a distinctly better clinical outcome with a longer overall survival and recurrence-free interval after chemotherapy than sporadic cancers[21]. Notably, it has been observed that BRCA-positive Epithelial Ovarian Cancer patients have better outcomes than non-hereditary epithelial ovarian cancer patients [32], and advanced-stage hereditary cancer patients survive longer than nonhereditary cancer patients[33].

The identification of BRCA1 and BRCA2 genes in 1994 and 1995 respectively[34,35] has had a great impact on the understanding and management of sporadic and hereditary breast and ovarian cancers. Whilst the risk of breast and ovarian cancer in the general population is 10%-13% and 1.7%, respectively, studies indicate that a woman with mutations in either BRCA1 or BRCA2 carries a lifetime breast cancer risk of 80% and 20%-60% for developing breast cancer and ovarian cancer respectively[3,36]. In the case of BRCA1 gene mutation carriers, the cumulative risk of cancer by the age of 70 years ranges between 51% and 95% for breast cancer, and between 22% and 60% for ovarian cancer. Similar trends are also observed in BRCA2 carriers, as the risk ranges between 33% and 95% for breast cancer, and between 4% and 47% for ovarian cancer[37].

Women who have an increased risk of breast and ovarian cancer are advised to consider risk-reducing strategies; however, such methods vary in their effectiveness. These strategies include surveillance (breast self-examination, clinical breast examination, screening using mammography and breast magnetic resonance imaging (MRI), trans-vaginal ultrasound scanning and serum (CA125), chemoprevention and prophylactic surgery (salpingo-oophorectomy and/or mastectomy)[38,39]. Risk-reducing strategies have been shown to have associations with a gain in life expectancy in BRCA1/2 carriers. Moreover, extended life expectancy can range from between a few months to a few years, although this is ultimately dependent on the prophylactic intervention (Table 1).

**Table 1:** Life expectancy gains from cancer prevention strategies for BRCA1/2 positive women

Author and year	Mutation type	Type of prophylactic intervention vs. surveillance	Life expectancy gain (year)
Sonnenberg et al 1993	BRCA 1/2	Tamoxifen for 5 years	1.6 to 2.2
Schrag et al 1997	BRCA1/2	Bilateral Mastectomy Bilateral oophorectomy	2.9 to 5.3 0.3 to 1.7
Grann et al 1998	BRCA1/2	Bilateral oophorectomy Bilateral mastectomy Bilateral mastectomy and oophorectomy	0.4 to 2.6 2.8 to 3.4 3.3 to 6.0
Grann et al 2000	BRCA1/2	Bilateral oophorectomy Bilateral mastectomy Bilateral oophorectomy and mastectomy Chemoprevention with tamoxifen Chemoprevention with raloxifene	0.9 ( 95% CI: 0.4-1.2) 3.4 (95% CI: 2.7-3.7) 4.3 (95% CI: 3.6-4.6 ) 1.6 (95% CI: 1.0-2.1) 2.2 (95% CI: 1.3-2.8 )
Schrag et al 2000	BRCA1/2	Tamoxifen for 5 years Bilateral oophorectomy Contra-lateral mastectomy	0.4 to 1.3 0.2 to 1.8 0.6 to 2.1
van Roosmalen et al 2002	BRCA1/2	Bilateral mastectomy and oophorectomy Breast screening and bilateral oophorectomy Bilateral mastectomy with ovarian screening	High risk: 11.7. Medium risk 6.6 High risk: 9.5. Medium risk 5.3 High risk: 4.9. Medium risk 4.4
Armstrong et al 2004	BRCA1/2	Bilateral oophorectomy Bilateral mastectomy and prophylactic oophorectomy	3.34 to 4.65 5.49 to 7.63

The aim of this review is to provide an up-to-date analysis and to subsequently summarise the available literature in relation to risk-reducing strategies, with a keen focus on prophylactic surgery.

## Methods

MEDLINE and PubMed were used to search for relative articles. Articles identified using the key words "breast cancer", "BRCA1", "BRCA2", "ovarian cancer", "risk reducing", "prophylactic", "mastectomy", "oophorectomy" and "life expectancy". Cross referencing was performed to identify other articles not identified at the initial search.

## Results

### Surveillance

The efficacy of surveillance in BRCA1/2 mutation carriers is difficult to determine. Importantly, it has been suggested that some BRCA1/2 carriers will not develop cancer during their lifetime[40], although the identification of such individuals is currently impossible. The concept of surveillance is based on early detection of cancer rather than cancer prevention. It is suggested that women at a high risk of developing breast cancer should perform a monthly breast self-examination, and also undergo bi-annual clinical examination[41,42] and annual digital mammography. Owing to the fact that the sensitivity of mammography is significantly reduced in younger women because of their dense breast tissue and rapid development of breast cancer in BRCA1/2 mutations, the use of alternative and more sensitive imaging modalities, such as MRI at shorter intervals, has been recommended[42-44]. However, the malignant lesions in BRCA mutation carriers frequently have morphological characteristics which are commonly seen in benign lesions, such as a rounded shape or sharp margins. With this in mind, MRI has the ability to evaluate the enhancement pattern and kinetics, and hence enables the detection of characteristics suggestive of a malignancy[45]. The use of MRI in addition to digital mammography for surveillance in BRCA mutation carriers is considered to be essential in such women--even if their breast density is low[46]. Undoubtedly, surveillance is the least invasive option; however, such a method is associated with various negative consequences, such as increased anxiety, false reassurance, and unnecessary biopsies[47,48]. Although MRI is an effective surveillance modality in BRCA mutation carriers--especially in younger women--a significant proportion of women are still found to have node positive breast cancer at the time of diagnosis[49]. Furthermore, whilst regular surveillance in women at an increased familial risk of breast cancer is associated with a good clinical outcome if they carry BRCA2 mutations or no detectable mutation, the outcome in carriers of BRCA1 mutations is significantly worse, even in the instance that their tumours are diagnosed at an apparently early stage[27]. The impact of regular breast screening in this context on overall survival ultimately remains unclear, and further research is required in order to evaluate the effect of different breast-screening strategies according to the mutation type, type and frequency of screening modality, and age.

Importantly, annual ovarian surveillance--conducted by pelvic examination, trans-vaginal ultrasound scanning and serum CA125 measurement--in women at increased familial risk of ovarian cancer is found to be ineffective in detecting tumours at a sufficiently early stage, which substantially influences survival in BRCA1/2 mutation carriers[50-52]. Serum proteomics technology is currently being evaluated in the context of ovarian cancer detection and seems to be promising.

### Chemoprevention

It is well recognised that oestrogen plays an essential role in mammary carcinogenesis by exerting a carcinogenic effect (through metabolites) on cellular DNA and thereby promoting the growth of ER positive breast cancer[53]. Therefore, chemo-prevention strategies aim to target the oestrogen receptor signalling pathway or oestrogen synthesis. Selective oestrogen receptor modulators (SERMs) have been suggested as chemo-preventative agents in BRCA mutation carriers. In a subgroup analysis of the National Surgical Adjuvant Breast and Bowel Project (NSABP-P1) trial [54], Tamoxifen was found to reduce the incidence of breast cancer in healthy BRCA2 mutation carriers but not in BRCA1 carriers when started at the age 35 years or older[55]. These findings are not surprising given the fact that BRCA2-related breast cancers are largely ER-positive tumours, whilst BRCA1-related breast cancers are predominantly ER-negative[14,56,57]. Furthermore, in BRCA1 or BRCA2 carriers with breast cancer, Tamoxifen use was associated with the prevention of secondary and contra lateral breast cancer[58,59].

Prophylaxis with SERMs was estimated to prolong survival by 1.6 to 2.2 years in BRCA gene mutation carriers[60]. The ablation of the endometrial lining can be considered in BRCA mutation carriers who opt for chemoprevention with Tamoxifen in order to reduce the risk of endometrial tumours.

Targeting oestrogen synthesis is considered to be an alternative to SERMs as a chemo-preventative risk-reducing strategy in high-risk women. Aromatase inhibitors (AIs) have been shown to be superior to Tamoxifen in the treatment of ER-positive breast cancer and the prevention of contralateral breast cancer in postmenopausal women with fewer side effects. It is likely that AIs will have a prophylactic role to play in BRCA gene carriers who opt to have prophylactic oophorectomy (PO) and breast surveillance. However, this potential role requires further evaluation through prospective controlled trials, with the inclusion of quality of life issues and skeletal complications as secondary endpoints.

Based on the knowledge that the risk of ovarian cancer and mortality is reduced by 50% and 80% respectively in long-term users of oral contraceptive pills (OCPs)[61,62], OCPs are considered as potential preventative measures for women at high risk of developing ovarian cancer. The use of OCPs was associated with significant ovarian cancer risk reduction in a case control study when used for a period of 6 or more years by BRCA1/2 carriers[63]. However further research is required in order to assess the chemo-preventative role of OCP amongst high-risk subjects. Furthermore, their use is known to be associated with an increased risk of breast cancer[64]; hence, their use should be considered in carefully selected candidates.

The non-specific action of existing DNA damage reagents poses a problem regarding chemoprevention in BRCA women. Such agents have been found to cause a similar extent of damage in both BRCA1 mutant and wild-type cells. The recent discovery that PARP-1 inhibitors kill BRCA1/2 deficient cells with a high level of specificity subsequently opens up a potential therapy which looks very hopeful for BRCA1 cancer patients. A number of studies have illustrated that BRCA1/2 pre-cancerous cells are very sensitive to PARP inhibition, whilst cancer cells exhibit different responses to PARP inhibition under a variety of different conditions. Furthermore, it has been suggested that these differences in PARP sensitivity can be attributed to distinct phases in BRCA associated tumorigenesis. Work conducted by De Soto & Deng[65] demonstrates that, during the early stages of tumorigenesis, there is a clear sensitivity of BRCA mutant cells to PARP inhibition. This has led to the inference that BRCA-deficient cells in this early pre-cancerous state receive a permissive mutation which is sensitive to PARP-1 inhibitors. Such agents may prove effective in reducing breast cancer risk amongst BRCA1 and 2 carriers.

### Prophylactic Surgery

Risk-reducing surgery in BRCA1/2 gene mutation carriers includes prophylactic bilateral salpingo-oophorectomy (PBSO) and/or prophylactic bilateral mastectomy (PBM). The aim of surgery is to reduce the risk of cancer development and to reduce mortality.

Notably, extensive counselling should be offered to all women who consider prophylactic surgery, including a detailed explanation of the cancer risk, complications associated with surgery, aesthetic outcome, quality of life issues, and potential life expectancy gain. The decision to proceed with prophylactic surgery is largely patient-driven depending on whether the patient feels comfortable living with the estimated risk and how she values the psychosexual function of the breast and ovaries. It is observed that many women who undergo BRCA testing use these results to make clinical decisions; those who choose risk-reducing surgeries typically do so within months of receiving BRCA-positive results. Predictors of risk-reducing surgery uptake include age below 60 years, prior breast cancer, and utilisation of another risk-reducing surgery.

Since the establishment of the link between breast and ovarian cancers and BRCA1/2 genes mutations, in addition to the increasing availability of genetic testing, research groups were faced with many questions concerning the efficacy and usefulness of risk-reducing surgery. Earlier studies have more keenly focused on decisions concerning whether or when to undergo prophylactic surgery. The magnitude of the potential benefit fundamentally depends on the risk of cancer associated with specific mutations, the prognosis of the tumours in carriers of the mutations, and the extent to which relief of anxiety could result from surgical prophylaxis. These benefits were to be weighed against an array of potential costs, including surgical complications and the potential impacts of mastectomy or oophorectomy on a woman's self-image, as well as on their sexual and reproductive function[66].

#### Prophylactic bilateral mastectomy (PBM)

There are no randomised controlled trials which have previously examined the potential impacts of PBM on survival; therefore, evidence has been derived from retrospective and short follow-up prospective studies in addition to hypothetical mathematical models with variable estimates.

Earlier studies have utilised the Markov model[60], which was developed with the objective to determine the survival benefits in BRCA1/2 carriers who undergo different types of prophylactic surgery. In studies performed by Grann *et al*., it was concluded that a 30-year-old BRCA1/2 positive woman could prolong her survival by 0.9 years (95% probability interval, 0.4-1.2 years) by having bilateral oophorectomy, 3.4 years (2.7-3.7 years) by having bilateral mastectomy, and 4.3 years (3.6-4.6 years) by having both procedures compared with surveillance alone. Chemoprevention with Tamoxifen and raloxifene was estimated to increase survival by 1.6 years (1.0-2.1 years) and 2.2 years (1.3-2.8 years), respectively. These findings suggest that prophylactic surgery is associated with substantial survival benefits[67,68]. Other authors show that, compared with breast and ovarian screening, the average gain in life expectancy for 30-year-old carriers in the high- and medium-risk categories was 11.7 and 6.6 years respectively for combined prophylactic mastectomy and oophorectomy, 9.5 and 5.3 years respectively for breast-screening and prophylactic oophorectomy, and 4.9 and 4.4 years respectively for prophylactic mastectomy with ovarian screening. This indicates that combined prophylactic mastectomy and oophorectomy is the most effective strategy for prolonging life[69]. Furthermore, PO seems to be superior to BPM in terms of prolonging survival. Similar findings were reported by Schrag *et al*. [70]; however, the authors suggest that gains in life expectancy decline with age at the time of prophylactic surgery, and are minimal for 60-year-old women. Also, amongst 30-year-old women, oophorectomy may be delayed by 10 years with little loss of life expectancy.

Furthermore, in the case of carriers of BRCA1/2 mutations who subsequently developed breast cancer, it was found that, with the use of the Markov model and depending on the assumed penetrance of the BRCA mutation, probabilities associated with developing contra lateral breast cancer and ovarian cancer, dying from these cancers, dying from primary breast cancer, and the reduction in cancer incidence and mortality due to prophylactic surgeries and compared with surveillance alone, 30-year-old early-stage breast cancer patients with BRCA mutations showed a gain in life expectancy of approximately 0.4 to 1.3 years from Tamoxifen therapy, 0.2 to 1.8 years from PO, and 0.6 to 2.1 years from prophylactic contralateral mastectomy (PCM). The magnitude of these gains is believed to be least for those women with low-penetrance mutations (assumed contra lateral breast cancer risk of 24% and ovarian cancer risk of 6%) and greatest for those with high-penetrance mutations (assumed contra lateral breast cancer risk of 65% and ovarian cancer risk of 40%). Notably, older age and poorer prognosis from primary breast cancer further attenuate such gains [71]. Undoubtedly, estimates of life expectancy gain may help women and their physicians consider the uncertainties, risks, and advantages of such interventions, and may accordingly lead to more informed choices concerning cancer prevention strategies[71].

In recent years, a few cohort and case control studies have considered the effectiveness of prophylactic surgery. However, it is of great importance to mention that they differ in quality, with some having significant methodological flaws--mainly owing to selection bias and retrospective study design.

Prophylactic bilateral mastectomy reduces the risk of breast cancer in BRCA mutation carriers by 85%-100%[72-74]. In a report published by Rebbeck *et al*., 483 women with BRCA1/2 mutations were studied. After a mean follow-up of 6.4 years, breast cancer was diagnosed in two (1.9%) of 105 women who had bilateral prophylactic mastectomy, and in 184 (48.7%) of 378 matched controls who did not have the procedure. The group therefore subsequently concludes that BPM reduces the risk of breast cancer in women with BRCA1/2 mutations by approximately 90%[74]. Furthermore, in a prospective study of 139 women with BRCA1 or BRCA2 mutations, after a mean follow-up of 3 years, breast cancer developed in 8 of 63 women who had elected surveillance, but in none of the 76 BRCA mutations carriers who had undergone prophylactic surgery[73]. Similarly, a report on an extended series with a longer follow-up of women with BRCA1/2 mutation having undergone PBM at the Rotterdam Family Cancer Clinic found that no primary breast cancer occurred after PBM after a median follow-up of 4.5 years, with one woman presenting metastatic breast cancer almost four years after her PBM where no primary was found[75].

It is accepted that no surgical technique for prophylactic mastectomy removes all breast epithelium; simple mastectomy removes approximately 95%-99% of breast glandular tissue; however, it causes a significant deformity, changes in body image and sexuality, as well as adverse effects on the psychosocial status of the affected woman, but not quality of life[76]. Moreover, it has been observed that most women opting for prophylactic mastectomy experienced significantly higher distress levels than mutation carriers who opted for surveillance as well as non-mutation carriers. This difference in levels of distress was found to be highest during pre- and post-test stages, and had almost disappeared during the one-year follow-up. Moreover, mutation carriers opting for prophylactic mastectomy are more often in their thirties, more often have young children, and also have a greater awareness of the genetic nature of cancer in the family than those opting for regular surveillance. Interestingly, their distress levels seem to significantly decrease 6 months or longer following surgery, possibly due to the significant risk reduction of developing breast cancer[77]. Furthermore, those undergoing mastectomy with delayed reconstruction report a lower impact on their self-esteem and sexual life versus those who only had the mastectomy[78].

Skin sparing mastectomy and immediate breast reconstruction (SSM & IMR) has been proved to be oncologically safe, and is associated with high satisfaction rate and low morbidity[79,80]. More recently, there has been increasing interest in offering BRCA1/2 carriers SSM and IBR, either by using an autologous flap, implant or both. On the other hand, SSM and IBR have many aesthetic advantages[81], and so it is fundamental to recognise that 5%-10% of breast tissue can be left in the subcutaneous flaps. This residual breast tissue may be a focus for breast cancer development in high-risk women with BRCA mutations; therefore, SSM or nipple-sparing mastectomy in risk-reducing surgery setting is feasible in most patients at an increased risk; however, it should be the subject of further prospective evaluations in order to ascertain its long-term oncological safety[82].

#### Prophylactic bilateral salpingo-oophorectomy (PBSO)

Since breast tumours are largely oestrogen-driven, it has been suggested that the hormonal blockade by oophorectomy inhibits the development of breast tumours[83]. Importantly, prophylactic oophorectomy has the advantage of preventing ovarian cancer as well as breast cancer. Prophylactic salpingo-oophorectomy, and the sectioning of both tubes and ovaries, is recommended in order to ensure occult carcinomas are not missed. Furthermore, since BRCA mutation carriers develop fallopian tube carcinoma and peritoneal papillary serous carcinoma, PBSO and sectioning both tubes and ovaries is therefore recommended in order to ensure occult carcinomas are not missed[31]. The demonstrated efficacy of oophorectomy in the case of BRCA mutation carriers is interesting, simply because most BRCA1-related breast tumours are negative for oestrogen receptors[9-11]. This observation can be attributed to the carcinogenic effect of oestrogen metabolites-DNA adducts[84].

In a large, retrospective analysis of 551 BRCA carriers, BSO was found to have reduced the risk of ovarian cancer by 96% and breast cancer by 53% at a mean follow-up of 9 years[85]. Similar findings were observed in a prospective study of 170 BRCA carriers. During a mean follow-up of 2 years, the incidence of ovarian or peritoneal cancer and breast cancer was significantly greater amongst those women who selected surveillance than amongst those who chose to undergo PBSO[86]. In a multicentre prospective study, Kauff *et al*. found that, during a 3-year follow-up, BPSO was associated with 85% reduction in BRCA1-associated gynaecologic cancer risk and 72% reduction in BRCA2-associated breast cancer risk. Although protection against BRCA1-associated breast cancer and BRCA2-associated gynaecologic cancer was suggested, neither effect reached statistical significance. The authors postulate that the protection conferred by PBSO against breast and gynaecologic cancers may differ between the carriers of BRCA1 and BRCA2 mutations[87].

Although several studies demonstrate that PBSO decreased the risk of both breast and ovarian cancers in BRCA1/2 mutation carriers[85-94], these studies were nevertheless heterogeneous and used different designs: whilst some were retrospective case control studies, others used a prospective cohort design. Even amongst prospective studies, the inclusion criteria and the definitions of follow-up times differed. In some studies, only unaffected mutation-positive women were included and followed up; whilst in others--particularly when examining ovarian cancer risk--women with breast cancer were included. Such differences in study design can introduce biases (such as survival bias), and can consequently have an impact on risk-reduction estimates.

Recently, Rebbeck *et al*. carried out a meta-analysis of ten studies which investigated breast or gynaecologic cancer outcomes in BRCA1/2 mutation carriers who had undergone PBSO. The authors subsequently concluded that PBSO was associated with a statistically significant reduction in breast cancer risk amongst BRCA1/2 mutation carriers. Similar risk reductions were observed in BRCA1 mutation carriers and in BRCA2 mutation carriers. Furthermore, PBSO was associated with a statistically significant reduction in the risk of BRCA1/2-associated ovarian or fallopian tube cancer; however, data were deemed insufficient to obtain separate estimates for ovarian or fallopian tube cancer risk reduction. Moreover, PBSO was strongly associated with reductions in the risk of breast, ovarian, and fallopian tube cancers, and should provide guidance to women in planning cancer risk-reduction strategies[95].

Furthermore, in a prospective study with a short-term follow-up, PBSO was associated with a 90% reduction in breast cancer-specific mortality, a 95% reduction in gynaecologic cancer-specific mortality, and a 76% reduction in overall mortality [89]. Such data demonstrate the utility of BSO in this population of patients given the fact that the procedure can be performed laparoscopically with minimal invasiveness, high efficacy, and low morbidity[96]. Chemoprevention with anti-oestrogens--especially in BRCA2 carriers--and breast surveillance can be added to PBSO in order to enhance its effective prophylactic role with a limited impact on quality of life.

### Limitations

This review paper provides an up-to-date analysis of our understanding of the advantages of risk-reducing strategies in BRCA carriers. Although the article summarises the evidence which suggests that different strategies are associated with varying degrees of cancer risk-reduction, such evidence is nevertheless derived from retrospective and short follow-up prospective studies in addition to hypothetical mathematical models. It is known that retrospective studies do not provide the highest level of evidence compared with randomised controlled studies; they are often less robust and usually carry a degree of bias. Furthermore, they tend not to be standardised in relation to confounding factors, such as patients' age, socio-economic class, and other factors.

Currently, there are no randomised controlled trials concerned with the demonstration of the potential benefits or harms of prophylactic surgery. This is due to different factors, such as the small number of BRCA mutation carriers, which necessitate multiple centres to participate. Conducting a randomised controlled trial in multiple centres poses potential problems, namely a lack of standardisation, which is the main issue as different surgeons have different surgical risk-reducing techniques. Furthermore, in light of the currently available evidence from retrospective studies, randomisation is deemed unethical, and women may therefore opt for risk-reducing surgery rather than observation.

## Conclusions

Selecting the most appropriate risk-reducing strategy is not a straightforward task. The impact of risk-reducing strategies on cancer risk, survival, and overall quality of life are the key criteria considered for making a good decision. There is no sole risk-reducing strategy which is able to fully meet all expectations and requirements in an individual woman. On the one hand, non-surgical procedures provide a good body image and quality of life, but may be associated with increased risk of advanced-stage cancer and mortality; on the other hand, surgery ensures a very high protection from cancer but is associated with a number of disadvantages of invasiveness, non-reversibility and surgical morbidity.

Based on the available literature, it is deemed appropriate to acknowledge that the clinical management of cancer risk in BRCA1 and BRCA2 mutation carriers is a rather complex and stressful task, which should consider individual patients' expectations and preferences. Such preferences can be informed by accurate knowledge of the risks and benefits of the interventions considered. Available studies confirm the essential role of PBSO in reducing the risk of both breast and ovarian cancer. Furthermore, other options for risk reduction should be considered, such as chemoprevention and risk-reducing mastectomy[74]. Although coordinated, PBM and PBSO are feasible procedures with acceptable morbidity in selected high-risk patients[97]; the timing of prophylactic surgery is still a matter of paramount importance to both patients and clinicians to consider during the counselling process.

It is recommended that prophylactic surgery should be performed as soon as possible, simply because of the early development of cancer in BRCA mutation carriers. Although ovarian cancer rarely occurs in premenopausal women, it is advised that BRCA mutation carriers should undergo PBSO immediately after childbearing is complete in order to reduce the risk of early breast cancer development.

Prospective randomised controlled trials examining the various interventions in relation to the woman's age and type of mutation are needed. However, the randomisation of BRCA carriers to different arms of trials is an extremely difficult and rather impossible process given the current available evidence.

## Abbreviations

DCIS: Ductal carcinoma in-situ; MRI: Magnetic resonance imaging; SERMs: Selective oestrogen receptor modulators; AIs: Aromatase inhibitors; PO: Prophylactic oophorectomy; OCPs: Oral contraceptive pills; PBSO: Prophylactic bilateral salpingo-oophorectomy; PBM: Prophylactic bilateral mastectomy; PCM: Prophylactic contralateral mastectomy; SSM: Skin sparing mastectomy; IBR: Immediate breast reconstruction

## Competing interests

The authors declare that they have no competing interests.

## Authors' contributions

MS performed literature search/review and manuscript writing. SB: helped in manuscript writing, KM involved in the concept, manuscript revision and writing. All authors read and approved the final manuscript.

## Pre-publication history

The pre-publication history for this paper can be accessed here:

http://www.biomedcentral.com/1472-6874/10/28/prepub
